# Building Friendships Through Volunteering in Late Life: Does Gender Moderate the Relationship?

**DOI:** 10.1093/geroni/igab046.2196

**Published:** 2021-12-17

**Authors:** Emily Lim, Changmin Peng, Jeffrey Burr

**Affiliations:** 1 University of Massachusetts Boston, Boston, Massachusetts, United States; 2 McCormack Graduate School, Boston, Massachusetts, United States

## Abstract

Friendship plays a crucial role in maintaining social connectedness in late life. Volunteering helps older adults to stay socially engaged and often times provides the opportunity to meet and make new friends. A small literature suggests that volunteering may be associated with friendship, but many studies are limited by reliance on small, non-probability samples and simplistic analytic approaches. The literature is also unclear on how volunteering behaviors relate to specific characteristics of friendships and whether there are gender differences that condition these relationships. Using the 2014 and 2018 waves of the Health and Retirement Study (N=1,638 ), we investigate whether volunteer status and hours volunteered in 2014 are associated with friendship characteristics in 2018 (i.e., number of close friends, friendship quality, and contact frequency) among community-dwelling adults aged 50 years and above (M=65.60 years old, SD=8.31). We also examine whether gender moderated these relationships. Volunteer status and hours in 2014 were positively associated with the number of close friends and contact frequency in 2018. Only those who volunteered 200 hours or more in 2014 were positively associated with friendship quality in 2018. Regarding gender differences, men who volunteered 200 hours or more in 2014 had higher friendship quality in 2018 than women, while women who volunteered 100-199 hours in 2014 had greater contact frequency in 2018 than men. Hence, our results suggest volunteering is integral in shaping late-life friendships and volunteering might be more critical for understanding friendship characteristics among older men and women.

